# Digital addiction and its relationship with cognitive function among children - A cross sectional study

**DOI:** 10.6026/973206300211025

**Published:** 2025-05-31

**Authors:** Karthika Devi Mariappan, Sasikala Palayan, Zealous Mary C., Joseph Jeganathan

**Affiliations:** 1 Department of Faculty of Nursing, Sri Ramachandra Institute of Higher Education and Research, Porur, Chennai, India; 2 Department of Paediatric Nursing, Indira College of Nursing, Thiruvallur, India; 3 Department of Nursing, College of Health and Sport Sciences, University of Bahrain, Kingdom of Bahrain

**Keywords:** Digital media, internet use, digital addiction, cognitive function, children

## Abstract

Modern children exhibit a significantly greater reliance on technology compared to earlier generation. Therefore, it is of interest to evaluate the digital addiction and its effect on cognitive function among children. A Cross sectional study using convenience sampling technique was used to collect data from 419 children aged 9-17 years from pediatric ward and Out Patient Department (OPD) in a tertiary care hospital. The analysis included 419 participants and examined the relationship between digital addiction, measured by the Digital Addiction scale for children (DASC), and cognitive function measured by the PedsQL Cognitive function scale. The mean DASC score was 61.41 ± 18.51, and the mean PedsQL score was 388.49 ± 138.17. A statistically significant negative correlation was found between DASC and PedsQL scores (r = -0.57, p < 0.001), indicating Digital addiction has negative impact on cognitive function of children. This suggests that as digital addiction increases, cognitive function tends to decrease.

## Background:

Smartphones were first introduced in 2007 and digital media has experienced an exponential change. With the introduction of readily portable devices [such as smart phones and tablets) and instantaneously available internet access, it has all but taken over children's lives. The first five years of life are critical for brain development and set the stage for the development of healthy habits and socio-emotional relationships [1]. Children have become highly dependent on technology when compared to the older generations. Children and adults of today are constantly attached to their electronics, this greatly affects their cognitive and physical development [2]. Over use of screens negatively impacts development across a number of domains, and as children get older, it can also lead to behavioral issues in preschoolers and school-age children [3]. Children are heavily engaged in digital activities, 20% spend over 5 hours daily on social media [4], 23% play video games almost every day, and 30% use electronic devices regularly [5]. Approximately 5% of these students displayed signs of addiction [6]. The research also noted a shift in online behavior over time: in 2013, 70% of adolescents used social media daily and by 2018, 45% were online "almost constantly" [7]. In rural school pupils in Haryana, India, behavioral addiction is highly prevalent [30.3%). Males are more likely than females to develop behavioral addictions to smartphones, personal phones, and other gadgets [8]. School children in rural India are becoming addicted to technology as a result increased mobile phone availability. Addiction is predicted by a few demographic and device-specific variables. It is possible that it can affect academic achievement of individuals due to addiction to technology. Larger-scale research is necessary for this, along with initiatives to encourage responsible gadget use [9]. According to global trend analyses, the rise in digital addiction in recent decades can be attributed to factors like internet accessibility in each country and the estimated amount of internet usage per person [10]. Internet addiction is becoming a major mental health issue in many countries. Research indicates that worldwide, around 6.0% of individuals between the ages of 12 to 41 may have Internet Use Disorder, with the Middle East having the highest prevalence. Additionally, a study covering 31 countries over three decades found that around 4.6% of adolescents aged 10 to 19 may have Internet Gaming Disorder [11]. There is a dearth of information regarding the detrimental impacts of youngsters being exposed to the newest digital media gadgets in our Indian society [12]. Therefore, it is of interest to study the Digital addiction and Cognitive function among the children, to correlate between Digital addiction and Cognitive function of children and to associate selected demographic variables with Digital addiction and Cognitive function of children.

## Methodology:

## Research design:

A descriptive, cross-sectional survey design was adopted to achieve objectives of the study.

## Setting:

The study was conducted in pediatric ward and Out Patient Department (OPD) in a tertiary care hospital, Tamilnadu.

## Participants and sampling:

A total of 419 children were selected using convenience sampling technique. The inclusion criteria are children aged between 9-17 years who visits the tertiary care Hospital, who possess their own digital devices and used it in their day-to-day life and children and their parents who are willing to participate in the study. Children with chronic medical or mental illness and physical or learning disability were excluded from the study.

## Data collection tools:

A correlational cross-sectional study design using a convenience sampling and adhered to the STROBE guidelines for reporting. Sociodemographic details such as: Age, gender, standard of education, place of living, usage of the device was collected. The study used two standardized scales: Digital Addiction scale for children (DASC) which consist of twenty-five questions with five-point Likert rating scale (always, often, sometimes, rarely, never) (Nazir Hawi, Maya Samaha,2019) and PedsQL Cognitive function scale using consists of six questions with a five-point Likert scale (almost always, often, sometimes, almost never). (Varni, 2011).

## Data collection procedure:

After obtaining Institutional Ethical Committee (IEC) and Heads of department, the purpose and procedure of the study was explained to the children and parents. Confidentiality and privacy were assured throughout the procedure. The tool was provided in English and Tamil. It took 15 -20 minutes to complete for each parent. Collected data were tabulated and analyzed.

## Data analysis:

Descriptive statistics (Mean, Standard Deviation) was used to assess the demographic variables, digital addiction and cognitive function. Karl-Pearson correlation used to identify the relationship between Digital addiction and cognitive function. Inferential statistics (Chi-Square Test) was used to determine the association between the demographic variable, digital addiction and cognitive function.

## Results and Discussion:

Among 419 participants, the demographic variables revealed that 58.7% belongs to 15-17 years of age and 55.1% are females. 27.2% of the samples were in secondary school and 73% of them are from urban place. 21.7% use digital devices for around 5-10 hours per day, 90% use smart phone devices and mostly for entertainment (66.8%). There is a significant association between duration of hours the device used and digital addiction (X2 =23.95, P<0.001) and Purpose of using the device with (X2=8.5, P<0.01). The association between cognitive function and demographic variables indicates there is a significant association between duration of usage and cognitive function (X2=7.9, P<0.01) and type of device used (X2 =11.89, P<0.01). Table 1 and Figure 1 - (see PDF) showed 52% of the study participants have mild digital addiction, 43.9% have moderate digital addiction and 4.1 % have severe digital addiction. Table 2 showed 37.2% have poor cognition level, 31.7% have moderate cognition level and 31% have good cognition level. Table 3 showed the mean score of digital addiction scale as 61.41 and cognition function score as 388.49. Table 4 indicates there is a negative correlation (r= -0.57, P<0.001) between digital addiction and cognitive level of the participants.

Upon analyzing the study with a focus on gender, it was found that on average, male children had higher levels of digital addiction compared to female children and this supported by Oktay and Ozturk. The trend may be attributed to societal norms that tend to be more tolerant of boys [13]. Children of age between 15 - 17 year old were severely addicted than the other age group. McCororie *et al.* (2020) stated that children between the ages of 10-14 year were also severely addicted. It is thought that 15 - 17 age group children were easily addicted because these age children were liked to be alone and active on social media [14]. This finding indicates that considering age is important when diagnosing and implementing interventions for digital addiction. Looking into the purpose of the device used, were mostly used for entertainment purpose only, which was supported by the study done by Celik (2023) [15]. Islam *et al.* (2020) identified that most of the children are on the internet use for the purpose of playing games. This similarity is thought to be due to the more entertainment app and online games [16]. Digital addiction, particularly due to fast-paced gaming or social media, has been linked to reduced attention spans and impaired memory retention. Prolonged digital media use alters the structure of brain regions associated with attention control, such as the anterior cingulate cortex [17]. Screen addiction can rewire young brains, reducing their capacity for sustained focus and deep learning [18]. Adolescents with high levels of Internet addiction showed impairments in cognitive flexibility, inhibitory control, and working memory [19]. The current study results reveals that 37.2 % children have poor cognitive function in relation with severe addiction of 4% among 419 samples. A study conducted by Liza *et al.* (2023) has stated prevalence of high gadget addiction and poor cognitive function of 46.9% and 46.5%, respectively and among the participants there is an association between gadget addiction and cognitive function [20]. The same was proved by Ahlam *et al.* (2023) that the cognitive function-attention domain accuracy has a statistically significant difference between smart phone addicted and non-addicted children [21].

## Conclusion:

Digital addiction presents a serious threat to cognitive health, impairing memory, and their overall performance. Adolescents who continuously rely on digital devices for entertainment may encounter with dependency issue and experience disruptions in their daily responsibilities. Hence, deliberate efforts are needed to regulate screen time and promote digital wellness which contributes for both personal and societal development.

## Recommendation for future research:

Future studies should aim to replication of this study in large samples to generalize the study findings. Future study would be extended to identify the children with poor cognition due to digital addiction and interventional strategies identified and evaluated its efficacy to reduce the digital addiction.

## Limitations:

This study mainly imitated to signal setting, which limited the ability to generalize the findings. The collected data was fully depended on self - reported measures of the children and parents.

## Funding:

Nil

## Figures and Tables

**Table 1:** Frequency and percentage distribution of level of digital addiction

**Level of Addiction**	**Frequency (n)**	**Percentage (%)**
Mild addiction	218	52
Moderate addiction	184	43.9
Severe addiction	17	4.1

**Table 2:** Frequency and percentage distribution of Level of cognition

**Level of cognition**	**Score**	**Frequency (n)**	**Percentage %**
Poor cognition	<331	156	37.2
Moderate cognition	<451	133	31.7
Good cognition	>450	130	31

**Table 3:** Mean and standard deviation of DAS and cognition score

**Scale**	**N**	**Mini**	**Maxi**	**Mean**	**Std. Deviation**
DAS Score	419	25	109	61.41	18.51
Cognitive Score	419	0	600	388.5	138.17

**Table 4:** Correlation between DAS and cognition score (N=419)

	**N**	**Mean**	**Standard Deviation**	**r- value**
				**p-value**
DAS Score	419	61.41	18.51	-0.57***
				<0.001
PedsQL score	419	388.49	138.17	
